# Bacterial Wilt in China: History, Current Status, and Future Perspectives

**DOI:** 10.3389/fpls.2017.01549

**Published:** 2017-09-11

**Authors:** Gaofei Jiang, Zhong Wei, Jin Xu, Huilan Chen, Yong Zhang, Xiaoman She, Alberto P. Macho, Wei Ding, Boshou Liao

**Affiliations:** ^1^Laboratory of Natural Products Pesticides, College of Plant Protection, Southwest University Chongqing, China; ^2^Jiangsu Provincial Key Lab for Organic Solid Waste Utilization, Jiangsu Collaborative Innovation Center for Solid Organic Waste Resource Utilization, National Engineering Research Center for Organic-Based Fertilizers, Nanjing Agricultural University Nanjing, China; ^3^State Key Laboratory for Biology of Plant Diseases and Insect Pests, Institute of Plant Protection, Chinese Academy of Agricultural Sciences Beijing, China; ^4^Key Laboratory of Horticultural Plant Biology, Ministry of Education, College of Horticulture and Forestry Sciences, Huazhong Agricultural University Wuhan, China; ^5^Key Laboratory of Potato Biology and Biotechnology, Ministry of Agriculture, College of Horticulture and Forestry Sciences, Huazhong Agricultural University Wuhan, China; ^6^Research Center of Bioenergy and Bioremediation, College of Resources and Environment, Southwest University Chongqing, China; ^7^Plant Protection Research Institute, Guangdong Academy of Agricultural Sciences Guangzhou, China; ^8^Shanghai Center for Plant Stress Biology, CAS Center for Excellence in Molecular Plant Sciences, Shanghai Institutes of Biological Sciences, Chinese Academy of Sciences Shanghai, China; ^9^Oil Crops Research Institute, Chinese Academy of Agricultural Sciences Wuhan, China

**Keywords:** bacterial wilt, China, distribution, host range, diversity

## Abstract

Bacterial wilt caused by plant pathogenic *Ralstonia* spp. is one of the most important diseases affecting the production of many important crops worldwide. In China, a large scientific community has been dedicated to studying bacterial wilt and its causative agent, *Ralstonia pseudosolanacearum* and *R. solanacearum*. Most of their work was published in Chinese, which has hindered international communication and collaboration in this field. In this review, we summarize the status of knowledge on geographical distribution, diversity, and host range of *Ralstonia* spp., as well as, the impact of bacterial wilt on important crops and disease control approaches, in China. We present areas of research and publications by Chinese scientists and propose the promotion of collaborative research within China and with the international community.

## Introduction

Bacterial wilt disease caused by *Ralstonia solanacearum* is a serious threat to crop production worldwide (Hayward, 1991). *R. solanacearum* forms a highly diverse species complex encompassing four phylotypes, five races and six biovars that have geographically distinct distribution (Fegan and Prior, 2005). Recent reports propose to separate *Ralstonia solanacearum* species complex into three species: *R. solanacearum* (phylotype II), *R. pseudosolanacearum* (phylotype I and II), and *R. syzygii* (phylotype IV) (Safni et al., 2014; Prior et al., 2016). This species complex infects 100s of plants, including many economically important crops, such as tobacco, tomato, and potato (Hayward, 1991). Even though different approaches have been developed to control this disease, we still lack an efficient and environmentally friendly control measure for most of the host crops.

To exchange knowledge and control strategies of bacterial wilt disease, several International Bacterial Wilt Symposia (IBWS) have been organized in different locations across the world including Taiwan (1992), Guadeloupe (1997), White River (2002), York (2006), Wuhan (2011), and Toulouse (2016). The recent 6^th^ IBWS held in July 2016 in Toulouse, France successfully brought together a community of researchers worldwide including agronomists, farmers, and private companies involved in the study and control of bacterial wilt. A total of 14 Chinese researchers from 6 institutions attended this symposium and presented their work on bacterial wilt. Even though bacterial wilt is becoming increasingly important in China, information exchange and coordinated research among different groups are relatively limited. Therefore, the First Chinese Bacterial Wilt Symposium was organized in Chongqing in December 2016 to bring together researchers to discuss the long-term strategies to understand and control bacterial wilt. This meeting was a landmark of bacterial wilt research in China and brought together both Chinese and international researchers.

Bacterial wilt is notoriously known in China as “Green wilt disease (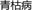, Qing Ku Bing)” by farmers and scientists because the leaves of the infected plant remain green when the plant starts to shows wilt symptoms. Before the 1960s, limited research in China was conducted on bacterial wilt disease, even though the disease was first recorded on peanut in the 1930s (Ma and Gao, 1956). Guangdong Academy of Agricultural Sciences conducted the isolation of *Ralstonia* spp. from many plants in Guangdong Province in the middle of the 1960s. With the intensification of agricultural production, the prevalence of bacterial wilt has increased on Solanaceous crops as well as other hosts. In recent four decades, extensive research on pathogenic aspects and disease management strategies has been carried out throughout the country. Here we summarized the history and current status of bacterial wilt, disease control approaches, and the research community of China. This article will provide basic information and some suggestions for further research on this aggressive disease.

## Distribution and Species Complexity

**FIGURE 1:**
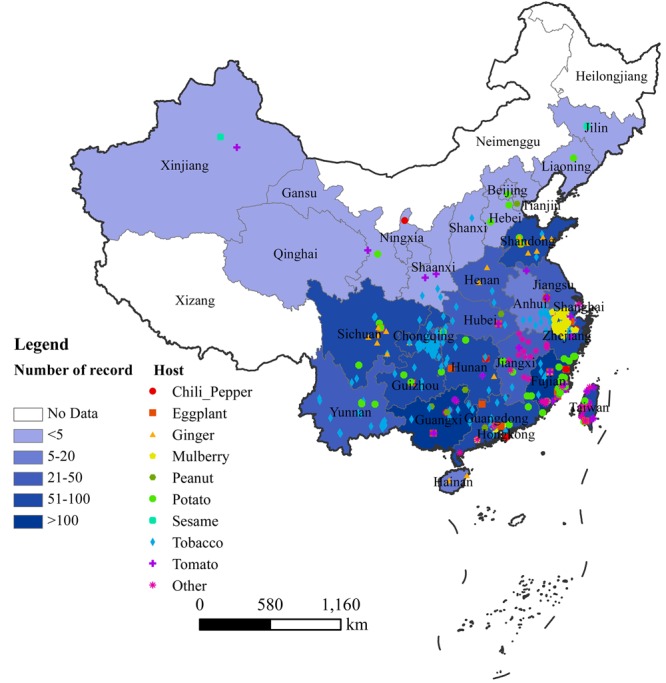
Geographical distribution of bacterial wilt in China. Records of each province were extracted from both CNKI and WoS, as well as information from website reports. Color depth indicates the number of records in each province.

In China, bacterial wilt disease has been reported in 30 provinces, with more in southern and eastern areas than northern and western areas (**Figure 1**). We could not find any published report of bacterial wilt occurrence in Tibet (Xizang) and Macau (**Figure 1**). There were sporadic reports from Shangai, Hongkong, Jilin, Xinjiang and Liaoning before 2012 (Fan, 1987; Huang, 1991; Liu W.Y. et al., 2012), but not recently (**Figure 1**). Fujian, Guangxi, Guangdong, Sichuan, Taiwan, Chongqing, and Hunan are the provinces where *Ralstonia* spp. strains have been most frequently isolated and described based on the available reports (**Figure 1**). Although *Ralstonia* spp. is prevalent in tropical and subtropical regions of the world, it has recently become a recognized problem in temperate regions as well (Elphinstone, 2005). The similar trend was observed in China. During the last decade, bacterial wilt disease has been reported more frequently in temperate and cool areas. The epidemics of bacterial wilt from warm lowlands (southern and eastern parts of China) to cool highlands of low and high latitudes of North China’s provinces is possibly due to the global climate warming, and changes in the cropping systems in China (Kong, 2003; Zhou et al., 2012; Liu et al., 2017).

Numerous studies have focused on the characterization of genetic diversity of *Ralstonia* spp. in China (Xu et al., 2009; Xue et al., 2011; Li et al., 2016; Liu et al., 2017). For instance, *Ralstonia* spp. infects more than 90 plant species in China (see Host Range). Some host plants such as peanut, potato, tobacco are commonly grown in more than 10 provinces and the topography and climate vary greatly between these agroecosystems (Chen and Zhang, 2000). The *Ralstonia* spp. strains from China belong to Race 1, 3, 4, and 5 and biovar 2, 3, 4, and 5 according to the race and biovar classification schemes (He et al., 1983; Hayward, 1991) (**Table 1**). Among those, Race 1 is most predominant being able to infect the largest number of hosts and being widely distributed across 17 provinces in China (**Table 1**). Overall, the evolutionary patterns of *R. solanacearum* in China are more divergent and complex than other parts of the world. This may be due to few countries have such a variety of environmental conditions for *Ralstonia* spp. as in China.

**Table 1:** Non-exhaustive list of the pathogenic *Ralstonia* spp. genetic diversity in China.

Phylotype	Sequevar	Host plant	Origin	Race	Biovar
I	12	Mulberry	Guangdong, Zhejiang	5	5
I	13	Balsam pear, Eucalyptus, peanut, tomato, potato	Guangxi, Shandong, Hunan, Fujian	1	3
I	14	Peanut, tomato, eggplant, pepper, ramie, beefwood, olive, ginger	Zhejiang, Sichuan, Fujian, Hubei, Guangxi, Guangdong, Hunan, Shandong, Taiwan	1	3, 4
I	15	Peanut, tobacco, tomato, eggplant, sweet potato	Guangxi, Fujian, Hubei, Hunan, Taiwan	1	3, 4
I	16	Tomato, eggplant, pepper, ginger	Fujian, Shandong, Henan, Jiangsu, Hubei	1	4
I	17	Peanut, tobacco, tomato, eggplant, pepper, potato, patchouli	Fujian, Hunan, Sichuan, Guangdong, Guangxi, Hubei, Guizhou, Chongqing, Yunnan, Shaanxi	1	3, 4
I	18	Peanut, tomato, potato, ginger, nightshade	Fujian, Sichuan, Henan	1	3, 4
I	34	Tobacco, tomato, eggplant, pepper	Fujian, Hunan, Taiwan, Guizhou, Jiangxi	1	3, 4
I	44	Peanut, tobacco, tomato, eggplant, Eucalyptus, ramie, ginger, beefwood, olive, patchouli, hibiscus, mulberry	Fujian, Guangxi, Guangdong, Sichuan, Hubei, Shandong, Shaanxi	1	3, 4
I	48	Tomato, eggplant, pepper, mulberry	Guangdong, Jiangsu, Zhejiang, Hunan, Hubei,	1	3
I	54	Tobacco	Chongqing, Yunnan, Guangxi	1	3
I	55	Tobacco	Yunnan	1	3
I	UN	Spinach, monk fruit, Chinese mesona, sesame	Guangxi, Guangdong, Jiangxi	1	3, 4
I	UN	Goatweed	Guangdong	4	3
II	1	Eggplant, potato, beefwood	Guangdong, Fujian, Hunan, Hubei, Yunnan, Guizhou, Shandong, Hebei, Beijing, Taiwan	2, 3	2
II	7	Tomato	Taiwan	UN	4

*UN, Unknown.*

Based on the phylotyping scheme (Fegan and Prior, 2005), global *Ralstonia* spp. strains comprise four phylotypes indicating their geographical origins. Each phylotype is further subgrouped into sequevars based on endoglucanase gene similarity (Fegan and Prior, 2005). The phylotype I strains reported in China comprise 15 sequevars and phylotype II strains are consisting of 2 sequevars, see **Table 1**. *R. pseudosolanacearum* strains possess high level of phylogenetic diversity, comprising a total of 10 sequevars (12 – 18, 34, 44, and 48) with the largest host range (Xu et al., 2009; Xue et al., 2011; Wang L. et al., 2017). Recently, new sequevars were identified including sequevar 14M (Wang L. et al., 2017) isolated from potato and peanut in 2015 and sequevars 54 (Li et al., 2016) and 55 (Liu et al., 2017) isolated from tobacco in 2016. *Ralstonia* spp. isolated from the same hosts, for instance tobacco (Zheng et al., 2007; Li et al., 2016; Liu et al., 2017), peanut (Xie et al., 2009) and mulberry (Huang et al., 2017), show high genetic diversity. For example, potato and tobacco are infected by 9 sequevars (1, 13 – 18, 34, and 14 M) and 10 sequevars (1, 13 – 18, 34, 44, 54, 55) (Xu et al., 2009; Xue et al., 2011; Li et al., 2016; Liu et al., 2017; Wang X. et al., 2017). Interestingly, the diversity of tobacco isolate phylotype I is negatively affected by the elevation, i.e., less divergent or more geographically distinct in the highland areas (Liu et al., 2017) possibly due to recent adaptation to cool temperatures. Further research is required to understand the molecular bases determining environmental adaption of this pathogen. Importantly, 91% of 123 potato *Ralstonia* spp. isolates from 13 provinces belong to phylotype II/sequevar 1, race 3 biovar 2, R3B2 (Wang X. et al., 2017). R3B2, known as cold-tolerant group, significantly constraints Solanaceous crops at higher land or in temperate zones of Africa, Asia, and Latin America (Champoiseau et al., 2009; Milling et al., 2009).

## Host Range

**FIGURE 2:**
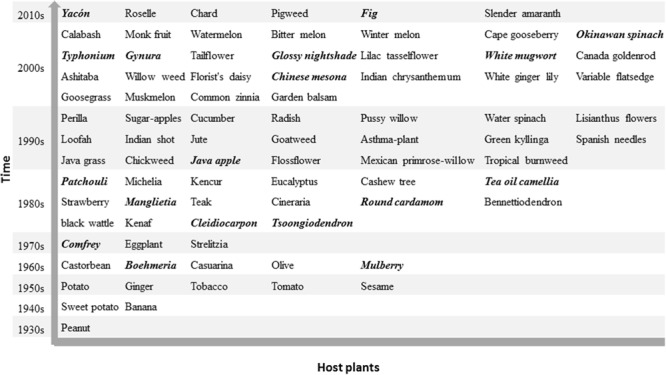
Host plants of the pathogenic *Ralstonia* spp. in China. A total of 84 plant species present here based on discovered decade since the first record on peanut in 1930s. Some of them are composed by more than one closed species like *Casuarina* spp., *Eucalyptus* spp. The common names in italic and bold indicate the host plants of *Ralstonia* spp. reported only in China. There is no ranked discovered time in this figure. Detail information of host plant and discovered year in Supplementary File 1.

The history of bacterial wilt in China started with the first report of an outbreak on peanut in the 1930s. Ralstonia spp. were then isolated from a sweet potato in 1946 (Hwang et al., 1956) as well as from ginger, sesame, potato, tobacco, and tomato in the 1950s (Wang, 1959; He et al., 1983). Since 1960s, diseased area has expanded greatly and many new host plants have been observed indicative of host range expansion. In addition to herbaceous plants, bacterial wilt also attacks several woody plants, including olive, mulberry, *Casuarina* spp. and *Eucalyptus* spp. For example, the olive bacterial wilt became an emerging bacterial disease in China when the olive was introduced from Albania to China in 1964 (Sichuan Academy of Forestry, 1977). Bacterial wilt of *C. equisetifolia* was discovered at Yangjiang and Baixian of Guangdong in 1964 (Liang and Wang, 1982), and later extended to southeast coast including Guangdong, Guangxi, Fujian, and Hainan after a typhoon in 1969 (Sun et al., 2013). Until now, the host range of *Ralstonia* spp. in China encompasses more than 90 plant species belonging to 39 botanical families with the largest number of hosts in Solanaceae (**Figure 2**). New hosts are still being reported including roselle and chard in Taiwan (Wu et al., 2013; Lin et al., 2015) and fig in Fujian (Jiang et al., 2016). Around 20 of these host plants have not been reported to become infected by *Ralstonia* spp. in other countries and are thus specific to China (Italic and bold highlight in **Figure 2**).

## Importance of Bacterial Wilt

Estimating economic losses caused by the bacterial wilt disease in China is difficult since direct yield losses vary widely according to host, cultivar, climate, soil type, cropping practice and pathogen strain. Therefore, the level of damage is commonly expressed on a crop-by-crop basis and can range from minimal crop loss to a very high economic damage. For instance, bacterial wilt of tomato is a severe problem in the southern provinces of the Yangtze River, ranging from 10% to 80% disease incidence depending on the crop seasons (Wei et al., 2011, 2015, 2017). In the case of potato, more than 10 provinces are infested by bacterial wilt disease with estimated yield losses ranging from 10–15% to even 80 or 100% in some hot-spot fields (Hua et al., 1985; Chen et al., 2005). The tobacco bacterial wilt broadly occurs in 14 out of the 22 main tobacco growing regions and has caused great economic losses in the recent years (Chen et al., 1997; Liu et al., 2017). Disease incidence is typically around 15–35%, but can reach up to 75% and even higher when associated with other root diseases such as Black shank caused by *Phytophthora nicotianae* var. *nicotianae*. In the wet and mono cropping tobacco areas, yield reduction ranges from 50 to 60% and up to even 100% during extreme outbreaks. With chili, disease incidence varies between 20 and 50% (Tan et al., 2014). Bacterial wilt is also prevalent in most ginger-growing areas, where it reduces the yield by 20–30% (Liu et al., 2005). Bacterial wilt of peanut has been observed in most of the 13 main peanut producing provinces. It is estimated to affect 800,000 hectares of agricultural land, which is nearly 16% of the total planting area in China. Yield losses in peanut varies between 10–20% and can reach up to 50–100% in extreme cases (Yu et al., 2011).

Bacterial wilt is also very serious in some woody and shrub plants, being especially significant in *Eucalyptus* spp. Since first reported on *E. saligna* and *E. grandis* in Guangxi in 1982 (Cao, 1982), this disease has been found in Guangdong, Yunnan, Hainan, Fujian, etc. (Wu and Liang, 1988). The bacterial wilt incidence of *Eucalyptus* spp. ranged from 20 to 40% in Guangdong and Hainan to 90% in Fujian (Wu et al., 2007). In beefwood (*Casuarina* spp.), bacterial wilt was first observed in Guangdong in 1964, and then was reported in Fujian, Guangxi, Hainan (Deng and Nan, 1979; Liang and Wang, 1982; Zheng et al., 1992). The bacterial wilt incidence of Beefwood varies around 50 and 90% (Sun et al., 2013). The bacterial wilt of mulberry was first found in 1973 at an orchard in Shunde of Guangdong (Lai et al., 1982), and expanded to Jiangxi in 1988 (Liu, 1996) and Zhejiang in 1992 (Chai and Dai, 1994). It was estimated that about 10% of the mulberry plantation was affected by bacterial wilt in China. The current outbreak in Zhejiang province has forced some growers to abandon mulberry cultivation, thus posing a serious threat to the local sericulture industry (Zhu et al., 2005; Pan et al., 2013). Therefore, bacterial wilt disease can be ranked as the most important disease in China due to its wide distribution and cumulative losses on many crops, trees and ornamental and medicinal plants.

## Disease Control

Cultural practices are traditional and popular approaches to control the bacterial wilt disease in China. Effect of crop rotation and grifting on bacterial wilt have been widely evaluated in greenhouse and field conditions (Huang et al., 1997, 2009; Zhang et al., 2007; Huang and Lei, 2013; Ouyang et al., 2015). Weeding and soil disinfection might relieve or extenuate bacterial wilt because contaminated weeds and soil are a major sources of *Ralstonia* spp. infections in the field (Fang et al., 2013). Soil amendment and fumigation are widespread means used in soil management in China. For instance, the formulated product S-H mixture (4.4% bagasse, rice 8.4%, 4.25% oyster shell powder, urea, 8.25%, 1.04% potassium nitrate, 13.16 and 60.5% silicate slag SSP) is effective to control several soil-borne diseases including fusarium wilt and bacterial wilt of many crops by enhancing the fertility and microbial abundance in soil (Sun and Huang, 1985; Yao et al., 1994). Calcium amendments like CaO and CaCO_3_ are effective in controlling bacterial wilt by inhibiting pathogen survival through changes in the pH and nitrite accumulation in the field (Gong et al., 2013; He et al., 2014). Bacterial wilt of tobacco can also be suppressed by supplementation of mineral nutrients like calcium and molybdenum (Zheng S. et al., 2014). Organic fertilizer and biochar amendments are promising alternatives to suppress bacterial wilt by increasing the soil pH, electric conductivity, organic carbon and nitrogen availability and microbial activities (Cai et al., 2003; Xue et al., 2010; Wei et al., 2011; Zhang et al., 2013; Liu et al., 2015; Gu et al., 2016). Soil fumigants like Chloropicrin, Dazomet, and Bromomethane (phased out in 2015 in China) can be applied to control bacterial wilt and other soil-borne diseases (Wang et al., 2010).

Breeding of crop cultivars with suitable resistance is regarded as a key approach for integrated management of bacterial wilt. Remarkable progress has been achieved in developing resistant cultivars for some economically important crops in China including peanut (Sun et al., 1981; Chen et al., 2007; Liao, 2014), tobacco (Liu Y. et al., 2012), potato (Deng et al., 2014), tomato (Yin et al., 2005), pepper (Dang et al., 2013), and eggplant (Li et al., 2014). The resistance levels and the diversity of germplasms are very important for the genetic breeding of bacterial wilt resistance. In addition, identification of quantitative trait loci (QTL) associated with the resistance has enabled breeders to develop resistant cultivars through marked assisted selection (MAS), such as *qBW-1* and *qBW-2* in peanut (Zhao et al., 2016) and *qBWR*-*3a*/-*3b* and *qBWR*-*5a*/-*5b* in tobacco (Qian et al., 2013). Many factors have limited the breeding of plant resistance against bacterial wilt including the difficulty to conduct large-scale screening of resistant materials, lack of elite resistant parents, undesirable genetic linkages between resistance and other agronomic traits as well as the high diversity of *Ralstonia* spp. virulence factors (Liao, 2005).

Biological control is another promising way to reduce bacterial wilt severity. Biological agents have been used to control bacterial wilt in China for a long time (Meng, 1964). In principle, any microbe which is able to inhibit *Ralstonia* spp. population density, or to reduce its pathogenicity, has the potential for biological control of bacterial wilt. The most frequently applied microbial agents are *Streptomyces* spp. (Lu et al., 2013; Xiong et al., 2014), *Bacillus* spp. (Ran et al., 2005; Lei et al., 2010; Wei et al., 2011; Wang et al., 2015), *Pseudomonas* spp. (Yang et al., 2008; Qiao et al., 2015; Hu et al., 2016), avirulent *Ralstonia* spp. mutants (Chen et al., 2004; Yang et al., 2008), phage (Wang X. et al., 2017) and other microbes (Guo et al., 2004; Xue et al., 2009; Yang et al., 2012; Huang et al., 2013). Due to the unstable performance of application of single biocontrol agent under field conditions (Wei et al., 2011, 2015, 2017), beneficial microbial consortia that could better utilize available resources and produce antibiotics may help to improve the consistency and efficacy of bacterial wilt biocontrol (Wei et al., 2015; Hu et al., 2016; Yang et al., 2017). Novel strategies are also being developed to enhance disease suppression. A recent study demonstrated that combination of antibiotic-producing *Bacillus* strains and *Ralstonia* spp. specific phages had a better control effect on bacterial invasion of tomato due to additive synergistic effects (Wang X. et al., 2017).

## Current Status of Bacterial Wilt Research in China

There have been three stages of the research on bacterial wilt in China. The first period (field practice) of research on bacterial wilt started with disease survey, pathogen identification in plant hosts, then extended to epidemiology and disease controls from the 1930s to the late 1970s. In second period, many researches had been moving from the field to laboratory, mainly on ecology, genetics, phytopathology, etc. This great increase was due to the improvement of education and the development of life science disciplines until the end of the 1990s. After that, the economic and scientific importance of bacterial wilt quickly gained attention across the international research community, prompting more scientists to dedicate to both fundamental and applied research on this topic. Meanwhile, the Chinese bacterial wilt research entered into a new era, i.e., the third period. It’s no longer simple field practice or laboratory research but from the field to the laboratory and back again. Researchers deploy practical strategies to cure the disease in the field, as it is exemplified by the extensive utilization of resistant cultivars in peanut and several Solanaceae crops, which has played an important role in reducing yield losses due to the disease.

**FIGURE 3:**
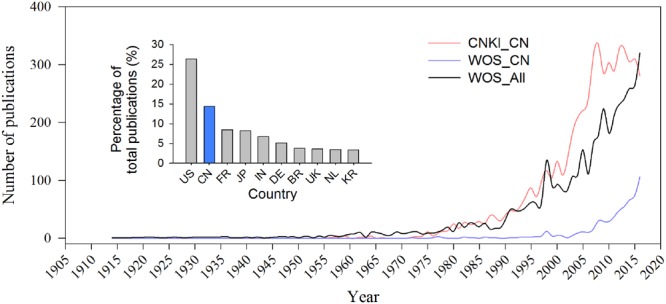
Publication records of the pathogenic *Ralstonia* spp. and bacterial wilt. Blue line indicates number of publications written in Chinese, i.e., records from CNKI. Red and black line show the records from Chinese organizations and world community on WoS. Small panel in **Figure 3** shows the Top 10 numbers of reports on *Ralstonia* spp. from WoS published by researchers from United States (US), China (CN), France (FR), Japan (JP), India (IN), Germany (DE), Brazil (BR), United Kingdom (UK), the Netherlands (NL), and Korea (KR).

Most publications related to bacterial wilt and *Ralstonia* spp. are written in Chinese, with 597 records from Web of Science (WoS) and 5441 records from China National Knowledge Infrastructure (CNKI) by the end of 2016. Only 50 publications were recorded in WoS but 1098 records in CNKI before the new century. Both records from WoS and CNKI have increased every year by following the trend of the world publication rate since the 1990s. The percentage of WoS publications from China increased from 1.64% (2001) to 14.42% (2016) and is now ranked now the second after the United States (**Figure 3**).

There are many organizations in China working on various research fields related to bacterial wilt and *Ralstonia* spp. biology. These areas include the ecology of the disease, pathogen diversity and evolutionary dynamics, microbiology and disease management and breeding of host-plant resistance. The main aims are described below:

(i) Ecology and diagnostics of bacterial wilt: assess the extent of problems caused by *Ralstonia* spp. to crops and understand the epidemiology of the disease in order to predict bacterial wilt outbreaks.
(ii) Genetic diversity and evolutionary dynamics of the pathogen: understand how host type and the environmental and geographic factors affect the evolution, diversification and population structure of *Ralstonia* spp.
(iii) Virulence mechanisms and host–plant responses: study pathogenicity determinants (e.g., type III/VI effectors) and complex regulation networks as well as the host responses upon *Ralstonia* spp. infections.
(iv) Control of bacterial wilt disease: delay or constrain bacterial wilt outbreaks and reduce yield losses by changing agricultural practices, developing chemicals and biocontrol agents, and breeding for resistant cultivars.

## Perspectives

In recognition of the importance of bacterial wilt in China, Southwest University, Nanjing Agricultural University, Institute of Plant Protection of CAAS, Institute of Tobacco Research of CAAS, Oil Crops Research Institute of CAAS and Municipal Agriculture Committee of Chongqing jointly organized the 1st China Plant Bacterial Wilt Symposium (CPBW) in Chongqing from 11th to 14th December 2016. Around 200 participants attended the meeting, including researchers and scientists from 67 organizations and 15 provinces. In addition, the Chairman of 2016 IBWS, Dr. Stéphane Genin, attended this meeting and highlighted the contribution of Chinese researchers to the global bacterial wilt community. In this congress, 40 speakers presented their works involving genetic diversity, evolution and virulence effectors of *Ralstonia* spp., mechanisms of interaction with host plants, significance of plant resistance and various approaches to biocontrol bacterial wilt.

The emerging problems of *Ralstonia* spp. in China are common to most regions in the world. These challenges include unusually broad host range, high and complex genetic diversity, strong and rapid pathogen adaption to new environments such as cold regions of high latitude and altitude. The main task of bacterial wilt research is coordinated on the basic knowledge of *Ralstonia* spp. and innovative control treatments in the fields. Seeking green, efficient, environmentally friendly and feasible strategies for the management of bacterial wilt is a great challenge to the international scientific community. Through in-depth knowledge exchange and discussion, the attendees at the 1^st^ CPBW reached a consensus and made a declaration toward the core research aims of the bacterial wilt research:

(i) Initiate a national network/platform for bacterial wilt research: develop collaborative innovations for cooperation and development, enhance communication and material exchanges, enable a concerted effort to jointly improve bacterial wilt research and favor the sustainable disease management.
(ii) Reinforcement of international exchanges and collaboration: strengthen cooperation in multidisciplinary fields and to maintain contributions to both theoretical and practical approaches.
(iii) Integrate production, education, and research: promote interactions between companies (application), universities (education) and research institutes (research) to find interdisciplinary solutions for the control of bacterial wilt.
(iv) Calling for policy guidance: gain attention from relevant governmental departments of China and other countries to support fundamental and applied research on bacterial wilt, call for special funding programs for the expansion of this research community, and maintain the high level of research.
(v) China Plant Bacterial Wilt Symposium: organize the meeting every 2 years by universities or institutes to reunite *Ralstonia* spp. research community. The 2nd CPBW will be hosted by Plant Protection Institute of CAAS in Beijing in 2018.

## Author Contributions

Organization: GJ and ZW; Curation: GJ, ZW, JX, HC, YZ, XS, AM, WD, and BL; Writing – original draft: GJ and ZW; Writing – review and editing: JX, HC, YZ, XS, AM, WD, and BL.

## Conflict of Interest Statement

The authors declare that the research was conducted in the absence of any commercial or financial relationships that could be construed as a potential conflict of interest.

## References

[B1] Cai Y. Liao Z. Zhang J. Kong W. He C. (2003). Effect of ecological organic fertilizer on tomato bacterial wilt and soil microbial diversities. *J. Appl. Ecol.* 14 349–353. 12836539

[B2] Cao J. (1982). Investigation report of bacterial wilt in *Eucalyptus saligna* and *E. grandis*. *Guangxi For. Sci.* 4 30–31.

[B3] Chai X. Dai H. (1994). Occurrence and control of Mulberry bacterial wilt in Zhejiang Province. *Bull. Seric.* 25 53.

[B4] Champoiseau P. G. Jones J. B. Allen C. (2009). *Ralstonia solanacearum* Race 3 biovar 2 causes tropical losses and temperate anxieties. *Plant Health Prog.* 10 10.1094/PHP-2009-0313-01-RV

[B5] Chen B. Jiang H. Liao B. Ren X. (2007). Progress on groundnut genetic enhancement for bacterial wilt resistance. *Chin. Agric. Sci. Bull.* 23 369–372. 10.1094/PHP-2009-0313-01-RV

[B6] Chen Q. Wong Q. Hu F. (2004). Effects of avirulent strains of *Ralstonia solanacearum* on tomato bacterial wilt. *Chin. J. Biol. Control* 20 42–44.

[B7] Chen R. Zhu X. Wang Z. Guo Z. Dong H. Wang L. (1997). A report of investigating and studying tobacco infectious diseases of 16 main tobacco producing provinces (regions) in China. *Chin. Tob. Sci.* 4 1–7.

[B8] Chen Y. He L. Xu J. (2005). Detection of bacterial wilt infection in potato using PCR. *J. Plant Prot.* 32 129–132.

[B9] Chen Z. Zhang X. (2000). Value of ecosystem services in China. *Chin. Sci. Bull.* 45 870–876. 10.1007/BF02886190

[B10] Dang F. Lei Y. Guan D. Wang Z. He S. (2013). Identification and evaluation of resistance to bacterial wilt in pepper. *Plant Sci. J.* 31 378–384. 10.3724/SP.J.1142.2013.40378

[B11] Deng H. Nan Y. (1979). Preliminary report on two strains of olive and peanut bacterial wilt. *Pract. For. Technol.* 10 19–22.

[B12] Deng R. Deng K. He T. Lei Z. Chen E. (2014). Progresses of main diseases resistance breeding in potato. *Southwest China J. Agric. Sci.* 27 1337–1342.

[B13] Elphinstone M. (2005). “The current bacterial wilt situation: a global overview,” in *Bacterial Wilt Disease and the Ralstonia Solanacearum Species Complex* eds Allen C. Piror P. Hayward A. C. (St. Paul, MN: APS Press) 9–28.

[B14] Fan H. (1987). Adjusting farmland microclimate to control the bacterial wilt in pepper. *Bimon. Xinjiang Meteorol.* 10 23.

[B15] Fang S. Gu G. Chen Y. Huang C. Chen S. (2013). Colonization and infection of *Ralstonia solanacearum* in weed roots. *Acta Tabacaria Sin.* 19 72–81. 10.1128/mBio.00875-13

[B16] Fegan M. Prior P. (2005). “How complex is the *Ralstonia solanacearum* species complex,” in *Bacterial Wilt Disease and the Ralstonia solanacearum Species Complex* eds Allen C. Prior P. Hayward A. C. (St. Paul, MN: APS Press) 449–461.

[B17] Gong M. Wang R. Du G. Zheng S. (2013). Effect of lime on the disease incidence of tobacco bacterial wilt. *Plant Dr.* 26 44–45.

[B18] Gu Y. Hou Y. Huang D. Hao Z. Wang X. Wei Z. (2016). Application of biochar reduces *Ralstonia solanacearum* infection *via* effects on pathogen chemotaxis, swarming motility, and root exudate adsorption. *Plant Soil* 415 269–281. 10.1007/s11104-016-3159-8

[B19] Guo J. H. Qi H. Y. Guo Y. H. Ge H. L. Gong L. Y. Zhang L. X. (2004). Biocontrol of tomato wilt by plant growth-promoting rhizobacteria. *Biol. Control* 29 66–72. 10.1016/S1049-9644(03)00124-5

[B20] Hayward A. C. (1991). Biology and epidemiology of bacterial wilt caused by *Pseudomonas solanacearum*. *Annu. Rev. Phytopathol.* 29 65–87. 10.1146/annurev.py.29.090191.000433 18479193

[B21] He K. Yang S. Y. Li H. Wang H. Li Z. L. (2014). Effects of calcium carbonate on the survival of *Ralstonia solanacearum* in soil and control of tobacco bacterial wilt. *Eur. J. Plant Pathol.* 140 665–675. 10.1007/s10658-014-0496-4

[B22] He L. Y. Sequeira L. Kelman A. (1983). Characteristics of strains of *Pseudomonas solanacearum* from China. *Plant Dis.* 67 1357–1361. 10.1094/PD-67-1357

[B23] Hu J. Wei Z. Friman V. P. Gu S. H. Wang X. F. Eisenhauer N. (2016). Probiotic diversity enhances rhizosphere microbiome function and plant disease suppression. *mBio* 7:e1790–16. 10.1128/mBio.01790-16 PMC5156302 27965449

[B24] Hua J. Zhang C. He L. (1985). A preliminary study on strains of *Pseudomonas solanacearum* of potato in China. *Acta Phytopathol. Sin.* 15 181–184.

[B25] Huang F. Chen Y. Zhou X. Li S. Li Y. Wu Z. (1997). Study on integrated control of tobacco bacterial wilt. *Guangxi Agric. Sci.* 1 32–35.

[B26] Huang J. Wei Z. Tan S. Mei X. Yin S. Shen Q. (2013). The rhizosphere soil of diseased tomato plants as a source for novel microorganisms to control bacterial wilt. *Appl. Soil Ecol.* 72 79–84. 10.1016/j.apsoil.2013.05.017

[B27] Huang T. Zhao X. A. Jiang X. (2009). Study on effect of different rootstocks on resistance to bacterial wilt in tomato. *J. Chang Veg.* 05x 55–56. 10.3865/j.issn.1001-3547.2009.10.021

[B28] Huang W. Zhang H. Xu J. Wang S. Kong X. Ding W. (2017). Loop-mediated isothermal amplification method for the rapid detection of *Ralstonia solanacearum* phylotype I Mulberry strains in China. *Front. Plant Sci.* 8:76 10.3389/fpls.2017.00076 PMC5281606 28197157

[B29] Huang Y. Lei D. (2013). Study on bacterial wilt resistance of tomato grafted by different rootstocks. *Acta Agric. Jiangxi* 25 73–75.

[B30] Huang Z. (1991). Discussion on the causes of cucumber bacterial wilt in suburbs of Shanghai. *Shanghai Veg.* 16 36–38.

[B31] Hwang L. Chen Y. S. Hwang H. Y. (1956). A preliminary study of sweet potato wilt and its control. *Acta Phytopathol. Sin.* 2 97–111.

[B32] Jiang Y. Li B. Liu P. Liao F. Weng Q. Chen Q. (2016). First report of bacterial wilt caused by *Ralstonia solanacearum* on fig trees in China. *For. Pathol.* 46 256–258. 10.1111/efp.12267

[B33] Kong F. (2003). Integrated control of tobacco bacterial wilt disease. *Tob. Sci. Technol.* 4 42–43.

[B34] Lai W. Jiang Z. Tan B. Wu G. Chen J. Guan W. (1982). Identification of Mulberry bacterial wilt pathogens. *J. South China Agric. Univ.* 3 66–73.

[B35] Lei J. Duan J. Ma H. Li J. Li H. Yang Z. (2010). Screening, identification and optimized fermentation condition of an actinomycete strain against *Pseudomonas solanacearum*. *Chin. J. Appl. Environ. Biol.* 16 79–83. 10.3724/SP.J.1145.2010.00079

[B36] Li W. Lv L. Wei C. (2014). Research advance in bacterial wilt resistance of eggplant. *Guangdong Agric. Sci.* 41 91–94.

[B37] Li Y. Feng J. Liu H. Wang L. Hsiang T. Li X. (2016). Genetic diversity and pathogenicity of *Ralstonia solanacearum* causing tobacco bacterial wilt in China. *Plant Dis.* 100 1288–1296. 10.1094/PDIS-04-15-0384-RE 30686189

[B38] Liang Z. C. Wang T. Z. (1982). Resistance measurements of *Casuarina equisetifolia* L. to bacterial wilt disease. *Trop. For.* 1 31–34.

[B39] Liao B. (2005). “A broad review and perspective on breeding for resistance to bacterial wilt,” in *Bacterial Wilt Disease and the Ralstonia solanacearum Species Complex* eds Allen C. Prior P. Hayward A. C. (St. Paul, MN: American Phytopathological Society) 225–238.

[B40] Liao B. (2014). “Peanut Breeding,” in *Genetics, Genomics and Breeding of Peanuts* eds Mallikarjuna N. Varsheney R. K. (Boca Raton, FL: CRC Press) 61–78.

[B41] Lin C. H. Chuang M. H. Wang J. F. (2015). First report of bacterial wilt caused by *Ralstonia solanacearum* on Chard in Taiwan. *Plant Dis.* 99 282–282. 10.1094/PDIS-07-14-0715-PDN 30699601

[B42] Liu D. (1996). How to control the bacterial wilt of Mulberry. *Seric. China* 17 36.

[B43] Liu L. Sun C. Liu S. Chai R. Huang W. Liu X. (2015). Bioorganic fertilizer enhances soil suppressive capacity against bacterial wilt of tomato. *PLOS ONE* 10:e0121304 10.1371/journal.pone.0121304 PMC4382293 25830639

[B44] Liu M. Zhang M. Ji J. Yin F. Zhang Y. Tu Y. (2005). Advances in research bacterial wilt of ginger in China. *Chin. Agric. Sci. Bull.* 21 337–340.357.

[B45] Liu W. Y. Chung K. M. K. Wong C. F. Jiang J. W. Hui R. K. H. Leung F. C. C. (2012). Complete genome sequence of the endophytic *Enterobacter cloacae* subsp. *cloacae* strain ENHKU01. *J. Bacteriol.* 194 5965 10.1128/JB.01394-1312 PMC3486125 23045485

[B46] Liu Y. Fan J. Li Y. (2012). Research progress on tobacco breeding resistant to bacterial wilt. *Acta Tabacaria Sin.* 18 93–99.

[B47] Liu Y. Wu D. Liu Q. Zhang S. Tang Y. Jiang G. (2017). The sequevar distribution of *Ralstonia solanacearum* in tobacco-growing zones of China is structured by elevation. *Eur. J. Plant Pathol.* 147 541–551. 10.1007/s10658-016-1023-1026

[B48] Lu Z. Peng L. Dng H. Zuo X. Peng J. Jiang X. (2013). Screening and identifying of antagonistic actinomycetes against *Ralstonia solanacearum*. *Chin. Tob. Sci.* 34 54–58.

[B49] Ma Q. C. Gao Y. C. (1956). Wilt disease of peanut. *Bull. Fujian Acad. Agric. Sci.* 2 89–98.

[B50] Meng X. (1964). A survey of peanut bacterial wilt. *Hubei Agric. Sci.* 3 37–41.

[B51] Milling A. Meng F. Denny T. P. Allen C. (2009). Interactions with hosts at cool temperatures, not cold tolerance, explain the unique epidemiology of *Ralstonia solanacearum* race 3 biovar 2. *Phytopathology* 99 1127–1134. 10.1094/PHYTO-99-10-1127 19740025

[B52] Ouyang X. Chao Z. Bai Z. Wu Y. Chen Q. Tian M. (2015). Preliminary study on grafting cultivation of tobacco with eggplant. *Hunan Agric. Sci.* 1 25–26. 10.16498/j.cnki.hnnykx.2015.05.010

[B53] Pan Z. C. Xu J. Prior P. Xu J. S. Zhang H. Chen K. Y. (2013). Development of a specific molecular tool for the detection of epidemiologically active mulberry causing-disease strains of *Ralstonia solanacearum* phylotype I (historically race 5-biovar 5) in China. *Eur. J. Plant Pathol.* 137 377–391. 10.1007/s10658-013-0249-9

[B54] Prior P. Ailloud F. Dalsing B. L. Remenant B. Sanchez B. Allen C. (2016). Genomic and proteomic evidence supporting the division of the plant pathogen *Ralstonia solanacearum* into three species. *BMC Genomics* 17:90 10.1186/s12864-016-2413-z PMC4736150 26830494

[B55] Qian Y. Wang X. Wang D. Zhang L. Zu C. Gao Z. (2013). The detection of QTLs controlling bacterial wilt resistance in tobacco (*N. tabacum* L.). *Euphytica* 192 259–266. 10.1007/s10681-012-0846-842

[B56] Qiao J. Chen Z. Liang X. Liu Y. Liu Y. (2015). Colonization of *Bacillus subtilis* Bs916 on tomato root. *Jiangsu J. Agric. Sci.* 31 229–234.

[B57] Ran L. X. Liu C. Y. Wu G. J. van Loon L. C. Bakker P. A. H. M. (2005). Suppression of bacterial wilt in *Eucalyptus urophylla* by fluorescent *Pseudomonas* spp. in China. *Biol. Control* 32 111–120. 10.1016/j.biocontrol.2004.08.007

[B58] Safni I. Cleenwerck I. De Vos P. Fegan M. Sly L. Kappler U. (2014). Polyphasic taxonomic revision of the *Ralstonia solanacearum* species complex: proposal to emend the descriptions of *Ralstonia solanacearum* and *Ralstonia syzygii* and reclassify current *R. syzygii* strains as *Ralstonia syzygii* subsp. syzygii subsp. nov., *R. solanacearum* phylotype IV strains as *Ralstonia syzygii* subsp. indonesiensis subsp. nov., banana blood disease bacterium strains as *Ralstonia syzygii* subsp. celebesensis subsp. nov. and *R. solanacearum* phylotype I and III strains as *Ralstonia pseudosolanacearum* sp. nov. *Int. J. Syst. Evol. Microbiol.* 64 3087–3103. 10.1099/ijs.0.066712-66710 24944341

[B59] Sichuan Academy of Forestry (1977). Study of bacterial wilt control on common olive. *Sci. Silvae Sin.* 13 61–66.

[B60] Sun D. R. Chen C. R. Wang Y. R. (1981). Resistance evaluation of bacterial wilt (*Pseudomonas solanacearum* E. F. Smith) of peanut (*Arachis hypogaea* L.) in the People’s Republic of China. *Proc. Am. Peanut Res. Educ. Soc.* 13 21–28.

[B61] Sun S. Wu H. X. Wang J. (2013). Research review on the bacterial wilt of *Casuarina*. *For. Pest Dis.* 32 29–34.

[B62] Sun S. K. Huang J. W. (1985). Formulated soil amendment for controlling *Fusarium* wilt and other soilborne diseases. *Plant Dis.* 69 917–920. 10.1094/PD-69-917

[B63] Tan Q. Q. Yuan J. Yang X. H. Chen X. Wang L. S. Wu S. P. (2014). Identification of resistance to Phytophtora blight and bacterial wilt in pepper varieties in Guizhou Province Regional Trial. *Seed* 33 82–85.

[B64] Wang D. Shen H. Ran L. (2015). Biocontrol of bacterial wilt in *Eucalyptus urophylla* and growth promotion by *Bacillus subtilis* strain CN181. *Hebei J. For. Orchard Res.* 30 331–334.

[B65] Wang H. Chen Y. Wang S. Li C. Su X. Li S. (2010). Control effects of chloropicrin soil fumigation on tobacco weeds and soil-borne diseases. *Chin. Agric. Sci. Bull.* 26 244–248.

[B66] Wang H. C. (1959). Banana diseases found in Taiwan. *J. Agric. For.* 8 117–166.

[B67] Wang L. Wang B. Zhao G. Cai X. Jabaji S. Seguin P. (2017). Genetic and pathogenic diversity of *Ralstonia solanacearum* causing potato brown rot in China. *Am. J. Potato Res.* 94 403–416. 10.1007/s12230-017-9576-2

[B68] Wang X. Wei Z. Li M. Wang X. Shan A. Mei X. (2017). Parasites and competitors suppress bacterial pathogen synergistically due to evolutionary trade-offs. *Evolution* 71 733–746. 10.1111/evo.13143 27925169 PMC5347860

[B69] Wei Z. Huang J. Yang T. Jousset A. Xu Y. Shen Q. (2017). Seasonal variation in the biocontrol efficiency of bacterial wilt is driven by temperature-mediated changes in bacterial competitive interactions. *J. Appl. Ecol.* 10.1111/1365-2664.12873 [Epub ahead of print]. PMC5638076 29081539

[B70] Wei Z. Huang J. F. Hu J. Gu Y. A. Yang C. L. Mei X. L. (2015). Altering transplantation time to avoid periods of high temperature can efficiently reduce bacterial wilt disease incidence with tomato. *PLOS ONE* 10:e0139313 10.1371/journal.pone.0139313 PMC4595502 26441225

[B71] Wei Z. Yang X. Yin S. Shen Q. Ran W. Xu Y. (2011). Efficacy of *Bacillus*-fortified organic fertiliser in controlling bacterial wilt of tomato in the field. *Appl. Soil Ecol.* 48 152–159. 10.1016/j.apsoil.2011.03.013

[B72] Wu Q. Liang Z. (1988). Identification and pathogenic tests of the causal organism of the bacterial wilt of *Eucalyptus*. *J. South China Agric. Univ.* 9 59–67.

[B73] Wu Y. F. Cheng A. S. Lin C. H. Chen C. Y. (2013). First report of bacterial wilt caused by *Ralstonia solanacearum* on Roselle in Taiwan. *Plant Dis.* 97 1375–1375. 10.1094/PDIS-02-13-0186-PDN 30722138

[B74] Wu Z. H. Xie Y. J. Luo L. F. Zhang W. Y. (2007). Advances in research on bacterial wilt Caused by *Ralstonia solanacearum* in *Eucalyptus* spp. in China. *For. Res.* 20 569–575.

[B75] Xie S. Ruan H. Du Y. Lin L. Ma H. Wang W. (2009). Genetic diversity of *Ralstonia solanacearum* on peanuts in Fujian. *Fujian J. Agric. Sci.* 24 351–354.

[B76] Xiong S. Sun C. Shi C. Jiang X. Peng L. (2014). Screening and Identifying of antagonistic actinomycetes against *Ralstonia solanacearum* in tomato. *North. Hortic.* 5 114–117.

[B77] Xu J. Pan Z. C. Prior P. Xu J. S. Zhang Z. Zhang H. (2009). Genetic diversity of *Ralstonia solanacearum* strains from China. *Eur. J. Plant Pathol.* 125 641–653. 10.1007/s10658-009-9512-9515

[B78] Xue F. Yan T. Yang L. Qiao J. (2010). Influences of organic fertilizer application on soil biological properties. *Chin. J. Eco Agric.* 18 1372–1377. 10.3724/SP.J.1011.2010.01372

[B79] Xue Q. Y. Chen Y. Li S. M. Chen L. F. Ding G. C. Guo D. W. (2009). Evaluation of the strains of *Acinetobacter* and *Enterobacter* as potential biocontrol agents against *Ralstonia* wilt of tomato. *Biol. Control* 48 252–258. 10.1016/j.biocontrol.2008.11.004

[B80] Xue Q. Y. Yin Y. N. Yang W. Heuer H. Prior P. Guo J. H. (2011). Genetic diversity of *Ralstonia solanacearum* strains from China assessed by PCR-based fingerprints to unravel host plant- and site-dependent distribution patterns. *FEMS Microbiol. Ecol.* 75 507–519. 10.1111/j.1574-6941.2010.01026.x 21204873

[B81] Yang T. Wei Z. Friman V. P. Xu Y. Shen Q. Kowalchuk G. A. (2017). Resource availability modulates biodiversity-invasion relationships by altering competitive interactions. *Environ. Microbiol.* 19 2984–2991. 10.1111/1462-2920.13708 28229529

[B82] Yang W. Xu Q. Liu H. X. Wang Y. P. Wang Y. M. Yang H. T. (2012). Evaluation of biological control agents against *Ralstonia* wilt on ginger. *Biol. Control* 62 144–151. 10.1016/j.biocontrol.2012.05.001

[B83] Yang Y. Liu J. Yang C. Gong H. Feng D. Xie B. (2008). Control of solanaceae vegetable bacterial wilt with avirulent hrp-mutants. *Acta Phytophylacica Sin.* 35 433–437.

[B84] Yao G. Zhang F. Li Z. (1994). Control of bacterial wilt with soil amendment. *Chin. J. Biol. Control* 10 106–109.

[B85] Yin G. Wang X. Zhang Y. Pan G. Yang Q. (2005). Research progress on tomato bacterial wilt and resistance breeding in China. *J. Yunnan Agric. Univ.* 20 163–167.

[B86] Yu S. L. Wang C. T. Yang Q. L. Zhang D. X. Zhang X. Y. Cao Y. L. (2011). *Peanut Genetics and Breeding in China.* Shanghai: Shanghai Science and Technology Press.

[B87] Zhang P. Wei Z. Zhu Z. Gao X. Deng K. Ran W. (2013). Effect of a bio-organic fertilizer on microbial flora and *Ralstonia solanacearum* population in rhizosphere soils of continuous cropping tomato and pepper. *J. Nanjing Agric. Univ.* 36 77–82.

[B88] Zhang Y. Huang Z. Guan G. Ding W. Tang Y. Li J. (2007). Influence of different agricultural ecological regulation and control measures on tobacco bacterial wilt. *Chin. Tob. Sci.* 28 49–52.

[B89] Zhao Y. Zhang C. Chen H. Yuan M. Nipper R. Prakash C. S. (2016). QTL mapping for bacterial wilt resistance in peanut (*Arachis hypogaea* L.). *Mol. Breed.* 36 13 10.1007/s11032-015-0432-0 PMC4735223 26869849

[B90] Zheng H. Lin J. Gao Y. Guo W. Huang R. (1992). Preliminary study on the resistance of common *Casuarina equisetifolia* to bacterial wilt and its physiological and biochemical mechanism. *J. Fujian For. Sci. Technol.* 1 9–13.

[B91] Zheng S. Ding W. Du G. Yang L. Liu X. Zhang Y. (2014). Control efficacy and action mechanism of mineral nutrition on tobacco bacterial wilt. *Sci. Agric. Sin.* 47 1099–1110.

[B92] Zheng X. Deng H. Liu Q. Chen Z. Chen Y. Li H. (2007). Strains and genetic diversity of *Ralstonia solanacearum* isolated from tobacco in Guangdong Province. *J. Huazhong Agric. Univ.* 26 463–468.

[B93] Zheng X. Song B. Tan X. Chen H. (2014). Identification of potato bacterial wilt pathogens. *Chin. Potato J.* 28 83–89. 10.1371/journal.pone.0096027

[B94] Zhou X. Wang J. Yang Y. Zhao T. Gao B. (2012). Advances in tobacco bacterial wilt disease. *Microbiol. China* 39 1479–1486.

[B95] Zhu Y. Ye Z. Lu Z. Wu Y. (2005). Research progress on *Pseudomonas solanacearum* S. *Dowson. Bull. Seric.* 36 6–9.

